# Switching VO_2_ Single Crystals and Related Phenomena: Sliding Domains and Crack Formation

**DOI:** 10.3390/ma10050554

**Published:** 2017-05-19

**Authors:** Bertina Fisher, Larisa Patlagan

**Affiliations:** Physics Department, Technion, Haifa 32000, Israel; Larisa@physics.technion.ac.il

**Keywords:** insulator–metal-transition, Joule-heating-induced-switching, electric current-induced self-heating, negative-differential-resistivity (NDR), Seebeck coefficient, Peltier coefficient

## Abstract

VO_2_ is the prototype material for insulator–metal transition (IMT). Its transition at T_IMT_ = 340 K is fast and consists of a large resistance jump (up to approximately five orders of magnitude), a large change in its optical properties in the visible range, and symmetry change from monoclinic to tetragonal (expansion by 1% along the tetragonal *c*-axis and 0.5% contraction in the perpendicular direction). It is a candidate for potential applications such as smart windows, fast optoelectronic switches, and field-effect transistors. The change in optical properties at the IMT allows distinguishing between the insulating and the metallic phases in the mixed state. Static or dynamic domain patterns in the mixed-state of self-heated single crystals during electric-field induced switching are in strong contrast with the percolative nature of the mixed state in switching VO_2_ films. The most impressive effect—so far unique to VO_2_—is the sliding of narrow semiconducting domains within a metallic background in the positive sense of the electric current. Here we show images from videos obtained using optical microscopy for sliding domains along VO_2_ needles and confirm a relation suggested in the past for their velocity. We also show images for the disturbing damage induced by the structural changes in switching VO_2_ crystals obtained for only a few current–voltage cycles.

## 1. Introduction

Many decades after its discovery [1,2,3], the steep and fast insulator–metal transition (IMT) of VO_2_ at T_IMT_ = 340 K remains one of the most attractive topics of research in condensed matter. To emphasize the importance of VO_2_ and its potential applications, the reader is directed to a rather incomplete collection of recent articles that address this topic [4,5,6,7,8,9,10,11]. Here we focus on the I–M domain structure in the mixed state of VO_2_ crystals during switching and to the damage caused by the structural changes [12]—both visible under the microscope due to the optical changes [13]. Twin boundaries of the insulating state are visible under the microscope due to polarization of the reflected light [14]. These twin boundaries disappear in the metallic state. Metal–insulator (semiconductor in the old notation) static or sliding domain patterns in the mixed state induced by heating [15] or self-heating [16] (upon passage of an electric current through the sample) have been recorded in movies. The most interesting dynamic pattern recorded in the seventies by one of us (Bertina Fisher) on a single crystal under constant current was of narrow semiconducting domains that nucleate at the positive edge of a static metallic domain and propagate towards the negative edge of the sample (in the sense of the applied current) [16]. The proposed interpretation—based on Reference [17]—suggested that sliding of the semiconducting (S) domains is driven by the Peltier effect at the two oppositely biased metal–semiconductor (MS) and semiconductor–metal (SM) interfaces [18]. Sliding in the conventional positive sense of the current is due to VO_2_ being an *n*-type semiconductor (its Seebeck and thus its Peltier coefficient are negative). According to this model, Bertina Fisher suggested that the maximal velocity of the semiconducting domains (*u*_max_) is obtained when the Peltier heat is fully exchanged with the latent heat; then:

(1) $$u_{\max}=\frac{\Pi_{\mathrm{SM}}J}{L}$$

where Π_SM_ is the Peltier coefficient of the VO_2_ insulator (semiconductor)–metal couple, *J* is the current density, and *L* is the latent heat per unit volume for the IMT [18]. The measured velocity obtained there for one given current was smaller but close to the calculated *u*_max_. The sliding domains were accompanied by voltage oscillations [19]; each creation/annihilation of an insulating (semiconducting) domain caused an upwards/downwards jump of the voltage along the sample. Other interesting metal–insulator domain patterns consisted of chains of alternating metallic (dark) and insulating (bright) triangles [18]; these were often static or mobile with much lower velocity. A unique pattern found in a single sample was of stripes along the *c*-axis [20]. The topic of sliding domains remained dormant for several decades and was awoken in the nano-era [21,22,23]. In the respective reports, the drift of the M–I domain walls in pure or doped VO_2_ current-carrying nano-wires were again attributed to the Peltier effect, on the basis of the sense of the drift which was consistent with the sign of Π_SM_. Relaxation-oscillations of frequency (*f*) increasing linearly with current were obtained in a W-doped VO_2_ nano-wire shunted by a capacitor; *f* as high as 5 MHz was obtained in a 1 µm nano-wire which sustained a single sliding metal–insulator boundary [21]. The frequency depends on the domains’ velocities as well on the number of domains along the sample. Thus, the early single result of *u*(*J*) [18] remained solitary and called for additional attempts to confirm the validity of the old Equation (1).

Growth runs that produce VO_2_ single crystals—single domains in the insulating state—with shiny, perfect surfaces and dimensions suitable for studying sliding IM boundaries using optical microscopy are rare, but possible. Along the way, many needles were found in which Joule heating-induced switching caused cracks and breaks coinciding with rather erratic propagation of M–I walls and the formation of hot filaments. The damage caused by heating or self-heating in VO_2_ due to the large symmetry and volume changes is well known. This acute problem sheds doubt even on the metallic resistivity of freshly-grown crystals of VO_2_ because these crystals underwent at least one cooling-heating cycle through the transition [24]. We therefore decided to show some examples of the formation of cracks and breaks along with the impressive sliding domains. Three essentially identical growth runs (see Section 4) contained crystals with very different geometries and morphologies; D1 contained many thin, high-quality free-standing fibers; D2 contained platelets, many of them imperfect; V were of poor geometry, with multi-domain insulating patterns. Although sliding domains seem to be a common property of many of the switching VO_2_ single crystals investigated, their velocity, *u*, as function of current-density, *J*, could be measured in only a few from batches D1 and D2. The results confirm Equation (1). Crack formation was recorded during switching of crystals from batch V.

## 2. Results

### 2.1. R(T)

**Figure 1:**
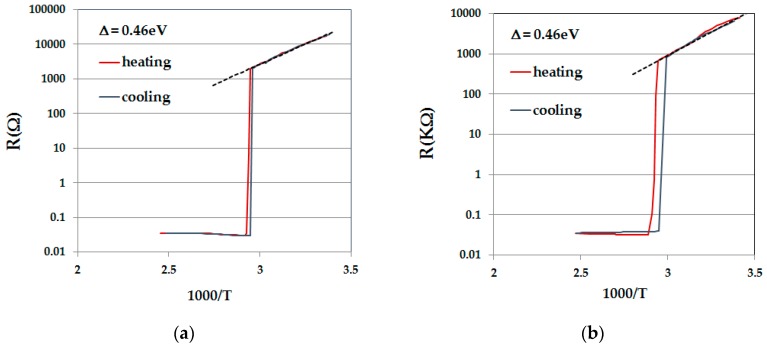
R(1/T) for samples (**a**) P4 (measured in the four-probe configuration) and (**b**) P2 (measured in the two probe configuration).

Figure 1a,b shows R(1/T) for two single crystals of VO_2_: P4 from batch V and P2 from batch D1 in the four-probe and two-probe configurations, respectively. The dimensions of P4 were: cross-section area A = 0.026 × 0.037 cm^2^ and the distance between voltage probes *d* = 0.12 cm; the dimensions of P2 were A ≈ 0.005 × 0.005 cm^2^ and Length l = 0.6 cm. The resistance jump at T_IMT_ is close to five orders of magnitude for P4 and more than four orders of magnitude for P2. The calculated resistivities (*ρ*) of P4 and P2 at 300 K are 120 and 220 Ω cm, respectively. The resistivity of P4 just below the transition (320 µΩ cm) is comparable with *ρ*_M_(IMT) = 220 µΩ cm documented in Reference [24]. The difference between the values of *ρ* (300 K) of P4 and P2 may be due in part to the uncertainty in the geometry of P2 (its small A) and to some damage; while P4 was a freshly grown single crystal, P2 underwent at least five I–V loops with switching events before the R(T) run. The activation energies of conduction (Δ)—which do not depend on geometry—are the same for the two samples, and they are in the range of the largest found in the literature, implying that conduction is intrinsic ([25] and the references within).

The I–V characteristics were measured in the two-probe configuration using amalgam dots for contacts. With these contacts, the samples are free to move, being held only by surface tension. The large resistance jump in Figure 1b indicates that the resistance of the amalgam dots is low enough, even for metallic VO_2_, and certainly for its mixed state.

### 2.2. I–V Characteristics and Sliding Domains

I–V measurements were carried out at ambient temperature (RT) in the two-probe configuration. The measurements were carried out over many orders of magnitude of currents by varying the applied voltage and the resistance in series with the sample (the load resistance—R_L_). Upon increasing current, I(V) becomes non-linear due to self-heating, the voltage V reaches a maximum, and a current-controlled negative differential resistance (NDR) regime is obtained (see Appendix A). T_IMT_(VO_2_) is reached in a portion of the sample within the NDR regime, and the sample enters the mixed state.

**Figure 2:**
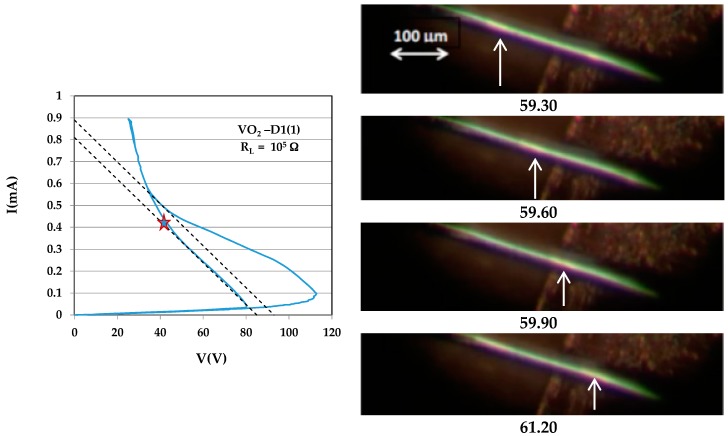
I–V characteristic of sample D1(1) and images of sliding domains snipped from a video 0.3 s apart at the point marked on the characteristic.

In search of sliding domains, we started with crystals with close-to-perfect morphology. The best-looking sample from series D1 was labeled D1(1). The I–V loop shown at the top of Figure 2 for crystal D1(1) (dimensions 0.005 × 0.005 × 0.5 cm^3^) is typical for switching of a high quality VO_2_ single crystal. The resistance in series with the sample (the load resistance—R_L_) for this loop is 100 KΩ. The initial resistance of the sample at ambient temperature (20 °C) is 4.5 MΩ. With increasing voltage, the I(V) trace becomes nonlinear due to self-heating; onset of the NDR regime occurs around 113 V. Upward switching between two steady states occurs along the upper dashed line, which represents the load line; the slope of the line is ≈2 × 10^−6^ A/V (inverse of R_L_). Above the end of this short segment of the load line, I(V) enters a perfectly reproducible range. At the highest current, the sample’s resistance is about two orders of magnitude lower than the R(RT) and less than one order of magnitude below R_I_(T_IMT_)—the sample is in a mixed state with a fraction of its volume in the insulating state. Backward switching occurs along the lower dashed line (parallel to the upper load line). Images of D1(1) were recorded on video during cycling through the I–V loops. Upward switching is marked by shuddering and twisting of the sample that often drive it out of focus, while downward switching is marked by a sudden freezing of motion. Sliding domains were observed for the whole range of the I–V loop between upward to downward switching. The sliding velocity increases with increasing current. The best microscope images were obtained for the slow domains (i.e., close to upward or downward switching); the contrast between the images of sliding domains and background becomes fuzzy when sliding is fast. Shuddering of the sample close to upward switching also disturbs the time-dependent image. The four frames shown in Figure 2 were snipped from the video at the scene that corresponds to the point on the loop shown above the frames. The current at that point was 0.4 mA. The images correspond to four instants 0.3 s apart (see times at right of images). Bright (insulating) domains move towards the right (in the sense of the current) in a blue (metallic) background. The domain at the left of the upper image covers a distance of ~200 µm (see right of the lowest image) over 0.9 s; the corresponding speed is *u* ≈ 0.022 cm/s. The current density *J* = 0.4/0.005^2^ = 16 A/cm^2^. The latent heat of the IMT of 1020 cal/mole [26] corresponds to *L* = 240 J/cm^3^. If these data fit Equation (1), then *uL/J* = 0.33 V ≤ Π_IM_(T_IMT_) = T_IMT_ S_IM_(T_IMT_), where S_IM_(T_IMT_) is the relative thermopower of the metal–insulator couple (S_I_–S_M_) at the transition. The above value of Π_IM_ corresponds to a rather high value of S_IM_; while S_M_ << S_I_, it is safe to take S_IM_ ≈ S_I_. S_I_(340 K) of −950 µV/K is rare in the literature, but comparable values were already measured in the past [18,26,27]. Π_IM_ (T_IMT_) was measured by two methods in Reference [17]; the value found there was 0.37 V.

Sliding domains were found in additional samples from the same series (D1), but no simple sequence could be obtained for measuring velocity. The samples were thinner than D1(1), and the pictures were blurred due to shuddering. In one case (sample D1(6) of dimensions 0.005 × 0.002 × 0.1 cm^3^), sliding domains were observed in a wide range of currents and in a few snipped pictures were sharp enough to distinguish shapes. No narrow domains were observed in this sample, and the patterns consisting of wide often of triangular shapes changed frequently, divided, or coalesced as function of time. This scenario repeated itself in a few additional samples from this batch. Figure 6c in [23] illustrates the development of triangular semiconducting domains from the metallic state near the compressed edge of a nanoplatelet.

**Figure 3:**
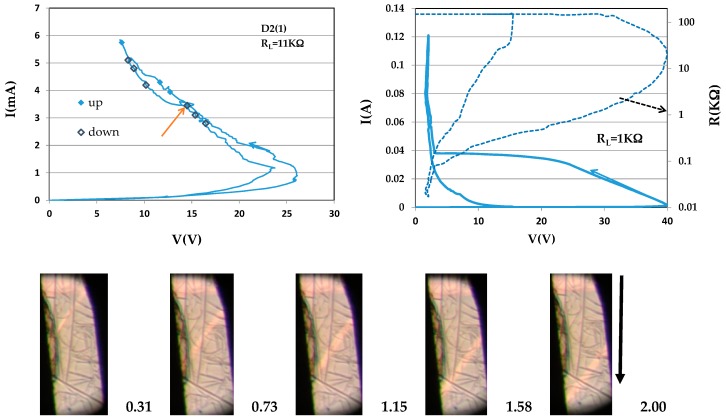
I–V characteristics for sample D2(1) with two load resistances. The images of sliding domains were snipped from the video 0.4 s apart at the point on the loop at left marked by the arrow. Additional sequences were obtained at the points marked on the loop for increasing and decreasing currents. The arrow marks the direction of the electric current.

Sample D2(1) (dimensions 0.018 × 0.016 × 0.2 cm^3^), although not perfect, enabled measuring *u*(*J*) for the various currents marked on the I–V loop at the left of Figure 3. The current was kept constant for a couple of minutes at each of the points marked on the loop in order to run the videos. This added roughness to the I–V characteristic in the NDR regime. Sequences of images were snipped from the videos to measure the velocity. The sequence shown in the figure corresponds to the current marked with an arrow on the I–V characteristic; the instances of snipping are 0.42 s apart. *u*(*J*) obtained for the various currents are summarized in Figure 7. I(V) (*y*-axis at left) and the corresponding log R(V) (*y*-axis at right) obtained in a range of much higher currents is shown at the right of this Figure. Steady state and reproducibility are reached at very low voltages when most of the sample is metallic. At the highest currents, the slope of I(V) is slightly positive (i.e., metallic-like). Upon backward switching, the resistance reaches its initial value; this unusually robust sample underwent this loop undamaged. The maximal change in the R(V) was more than four orders of magnitude. No sliding domains were observed in the range of currents of the loop at right. In a following attempt to increase the range of currents (by decreasing R_L_) the sample’s resistance became infinite—the sample collapsed to thermal runaway.

**Figure 4:**
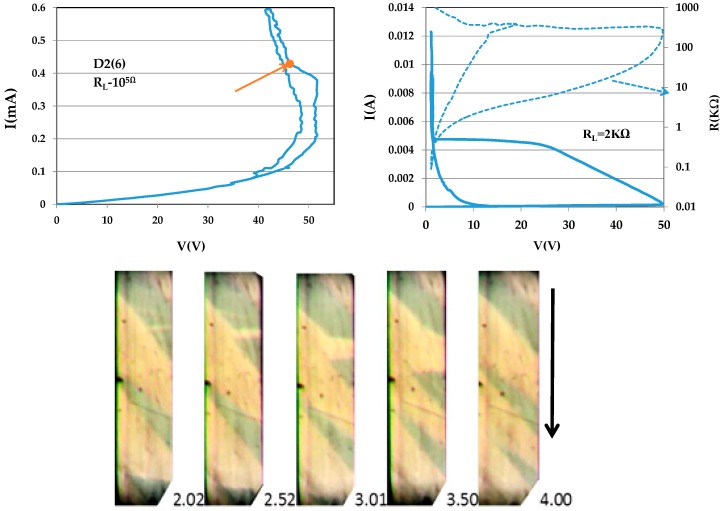
I–V characteristics for sample D2(6) with two load resistances. The images of metal (M, dark)–insulator (I, bright) domains snipped from the video 0.5 s apart at the point on the loop at left marked by the arrow. The pattern changes with time; the width of a barely visible narrow semiconducting domain increases with time, and the domain changes inclination.

Figure 4 shows I–V characteristics for sample D2(6) (dimensions 0.0138 × 0.0029 × 0.2 cm^3^) and five frames snipped from the video 0.5 s apart at the scene corresponding to the point on the loop at the top of Figure. This sample exhibits features similar to those of D1(6) (i.e., patterns that change with time). The width of a thin, horizontal, barely visible semiconducting domain in the first frame increases with time, the domain changes inclination, and after 2 s, becomes a thick domain at the expense of the neighboring domain. It is notable that the I–V characteristic shown at the right is very similar to that at the right of Figure 3.

**Figure 5:**
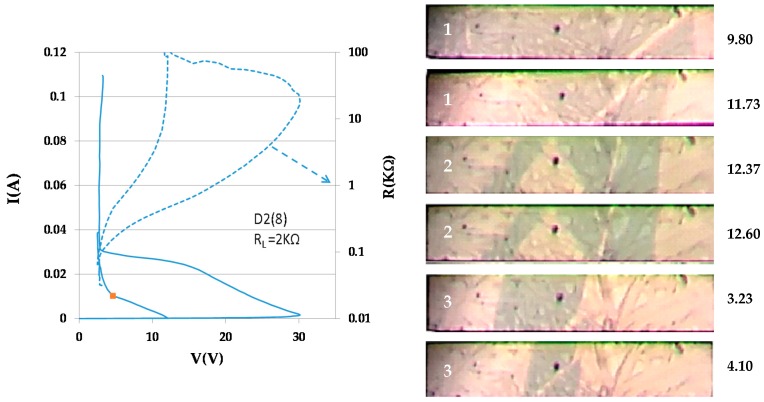
The first of three almost identical I–V characteristics of sample D2(8). The M–I patterns for the same current (see pairs of images at the right) are very different. A narrow semiconducting domain slides with very low velocity.

Several I–V loops were recorded for sample D2(8) (dimensions 0.019 × 0.009 × 0.15 cm^3^). Three loops (1, 2, and 3) for relatively high currents were practically identical with (V_max_(1) = 30.8 V, V_max_(2) = 30 V, and V_max_(3) = 29 V), respectively; for clarity, only the first loop is shown in Figure 5. Pairs of frames snipped from the videos at the scenes corresponding to the point marked on the first I–V characteristic, for the instants shown at the right of the frames are shown in the Figure. The M–I patterns of the three loops at the same current are very different. They have in common the appearance of a thin, slowly drifting semiconducting domain.

**Figure 6:**
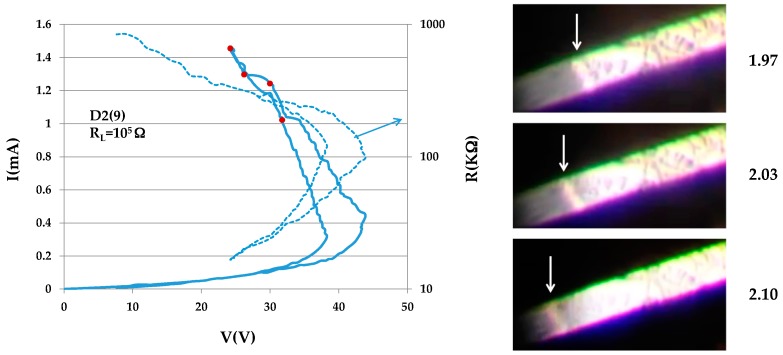
I–V characteristic of sample D2(9) and three images of a portion of the sample snipped 0.6 s apart.

Sample D2(9) (dimensions 0.0057 × 0.0045 × 0.2 cm^3^) with its rough matte surface was quite surprising. Sliding domains were observed over a wide range of currents above the switching threshold. On the loop shown in Figure 6, we stopped the current four times to take videos (see the I–V loop in Figure 6). Additional data were obtained for a second loop (not shown) which was similar but not identical. The domains were hardly visible within the rough portion of the surface; their rapid movement helped distinguish them from the noisy background. They became visible in a small portion of the sample that was smooth enough. A sequence of three snipped images, 0.1 s apart, are shown at the right of the I–V characteristic; these show a domain travelling in the smooth part of the sample. The domain shown here is perpendicular to the samples’ edges—a rather rare case. The inclinations of other domains observed in this sample are of the more frequent ±50°.

**Figure 7:**
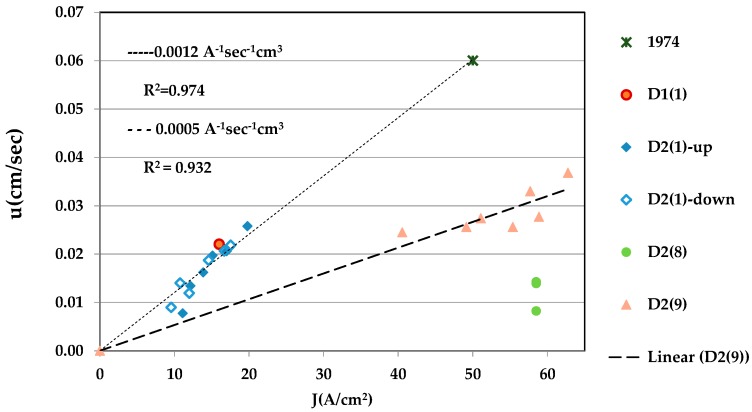
Sliding velocity as function of current density in samples of VO_2_.

The old and new data for the sliding velocity *u*(*J*) of narrow semiconducting domains are plotted in Figure 7. A linear trend-line was fitted to the data for the sample of 1974 [14], D1(1) (Figure 2), and D2(1) (Figure 3). The slope of the fitted line (≤ Π_SM_/*L* according to Equation (1)) is 0.0012 A^−1^·s^−1^·cm^3^. For *L* = 240 J/cm^3^, Π_SM_ ≥ 0.29 V, in good agreement with the values of Π_SM_ for pure VO_2_. Note that *u*(*J*) of D2(1) at low currents lie below the fitted line. The data of *u* for the narrow domains seen in sample D2(8) (Figure 5) lie far below the line (the lowest point is for Loop 1) of the series. The set of *u*(*J*) data for sample D2(9) lie in between. The slope of the fitted straight line to these data is 0.0005 A^−1^·s^−^1·cm^3^. This is probably related to the poor morphology of the sample.

Supplementary material S1 contains video clips for samples “1974”, D1(1), D2(1), D2(6), D2(8), and D2(9).

### 2.3. Crack Formation in Switching VO_2_ Crystals

**Figure 8:**
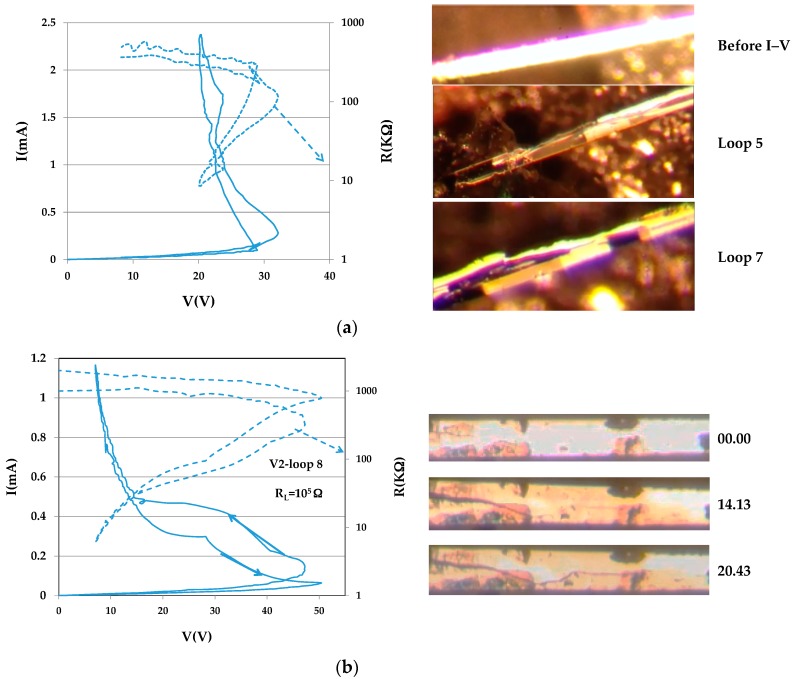
(**a**) The image of a portion of sample V1 before I–V cycling, an instant during the fifth loop when the cracks became visible (I–V at left) and during Loop 7; (**b**) A portion of sample V2 at three instances during switching in its eighth I–V loop (left).

Crack formation during switching is vivid and colorful in videos. These become non-interesting and even boring when turned into sets of still pictures. Nevertheless, the problem of damage in switching VO_2_ samples is too serious to be ignored. From a variety of videos, only a few instants from the troubled history of two samples were chosen. The two crystals are V1 (dimensions 0.010 × 0.0034 × 0.1 cm^3^) and V2 (dimensions 0.013 × 0.0078 × 0.152 cm^3^), and the chosen images are shown in Figure 8a,b. Figure 8a shows a portion of sample V1 before I–V cycling and during switching in the fifth I–V loop (left, when the cracks first became visible), and during the seventh loop. The dark portions of the sample are metallic. The maximal current density through the sample was 60 A/cm^2^, about one order of magnitude lower than the current densities in that left sample D2(1) intact. Figure 8b shows a portion of sample V2 at three instants during switching in its eighth I–V loop (left). The maximal current density in this sample was J_max_ ≈ m10 A/cm^2^, but the crack appeared at lower J. A few seconds later, the crack expanded to the right.

Supplementary material S2 contains video clips for samples V1 and V2.

## 3. Discussion

The detailed properties of materials are best studied in single crystals. In this work, we investigated VO_2_ crystals prepared according to the same protocol. Samples from three different batches had the same T_IMT_—a property regarded as very sensitive to the purity of the material—but behaved very differently upon switching to the mixed M–I state. This shows that very subtle details of the crystal growth which were not yet under control govern the quality of the products.

The main property investigated in this work is so far unique to switching VO_2_ single crystals: sliding semiconducting (SC) domains within a metallic background, in the conventional positive sense of the electric current. Moving boundaries were detected in all the samples of VO_2_ at currents above switching to the mixed M–I state. Narrow SC domains of constant shape and inclination sliding with constant velocity were scarce. Nevertheless, we were able to add data that confirm Equation (1). We suggest that the material property that governs sliding domains in VO_2_ samples is homogeneity at all scales (implying an absence of twin boundaries in the insulating state, or of any other defects). One cannot imagine a pair of parallel M–I and I–M barriers sliding as a solid entity if the current density is inhomogeneous.

Very large inhomogeneity of the current density is probably the cause of crack formation in crystals. This is also a warning signal for (polycrystalline) films of VO_2_ used in applications; films are more robust than the single crystals, but they may not be immune to the damage accumulated after cycling countless times through the transition. A significant effort is presently invested in developing strategies to avoid the physical destruction of VO_2_. Highly repetitive (>10^4^) cycling through the phase transition without structural damage was demonstrated in a nanocomposite of VO_2_ [28].

Thermally-, Joule-heating- or photo-induced resistive switching in materials exhibiting IMT above RT have many potential applications [29], but such materials are scarce and the high temperature at which the devices function affect their stability. One such material is Ca_2_RuO_4_; T_IMT_ of pure single crystals is 357 K, the resistance jump is less than one order of magnitude, and the magnitude of the crystal axes change is comparable to that of VO_2_ [30]. Single crystals shatter violently upon cycling through the transition, which makes the resistivity measurements a difficult task. From the large family of vanadium oxides exhibiting IMT, only VO_2_ and V_3_O_5_ have T_IMT_ > RT. T_IMT_(V_3_O_5_) = 428 K, the resistance jump is approximately two orders of magnitude, and the changes in symmetry and lattice parameters are small. Homogeneous heating of V_3_O_5_ samples up to 450 K—even for long durations—do not affect R(T). Self-heating by high currents induced significant changes in repeated I–V characteristics due to changes in oxygen stoichiometry [31]; the most dramatic effect was the reversible transformation of a small portion of the sample into VO_2_ and back to V_3_O_5_. These undesired changes were prevented by limiting the range of currents.

The static or dynamic ***natural*** M–I boundaries in the mixed state of VO_2_ obtained upon applying a thermal gradient on the sample around T_IMT_ or by passing an electric current through the sample provide an interesting and timely topic of investigation in the intense modern studies of surfaces and interfaces [32].

A final question is in order: are the sliding domains a property unique to VO_2_? Can such domains be found in a system with IMT below RT, such as V_2_O_3_?

## 4. Materials and Methods

Single crystals of VO_2_ of various shapes, needles or platelets of various sizes (the longer, the thinner), have been grown by self-flux-evaporation [16] from Aldrich 99.99% V_2_O_5_ powder with the addition of small quantities of VO_2_ crystallites. Three essentially-identical growth runs (D1, D2, and V) contained crystals with very different microstructures; D1 contained many thin high-quality free-standing fibers; D2 contained platelets, many of them imperfect; V were of poor geometry, with multi-domain insulating patterns. Nevertheless, R(T) of all three types of crystals proved that they were pure VO_2_. R(T) of the VO_2_ crystals was measured in the four-probe or two-probe configurations (with connected adjacent voltage and current probes). The four-probe configuration was employed for determination of the activation energy of conduction in the semiconductor close to IMT and the magnitude of the resistance jump. The contacts were indium-amalgam dots. These are ohmic contacts of low resistance for semiconducting VO_2_. The magnitude of the activation energies and the resistance jump at the IMT of two-probe and four-probe devices from these batches are usually comparable, the latter showing that the resistance of the amalgam dots is low enough even for metallic VO_2_ samples and certainly for samples in the mixed state. With these contacts, the samples are free to move, being held only by surface tension. I–V measurements were carried out at ambient temperature in the two-probe configurations. These measurements were carried out over many orders of magnitude of currents by varying the applied voltage and the load resistance. The I–V loops were recorded on a YEW type 3036 X-Y recorder while the samples were viewed under the microscope. Photos and videos were taken with the camera of an iPhone 5 attached to a Zeiss stereo microscope. Dynamic phenomena were processed using the Movie Maker Editor.

## Supplementary Materials

The following are available online at www.mdpi.com/1996-1944/10/5/554/s1. Video S1—Joined video clips for samples “1974”, D1(1), D2(1), D2(6), D2(8) and D2(9) showing sliding domains in the mixed M-I state induced by electric field. Video: ”1974” originates from an 8 mm Kodak movie, recently digitized. Video S2—Joined video clips for samples V1 and V2 showing crack formation during resistive switching induced by an electric field.

## Appendix A.—NDR due to Self-Heating

Below the IMT, VO_2_ has a negative temperature coefficient (NTC) of resistance (dR/dT < 0). The slope of R(T) is moderate in the activated (semiconducting) regime, and very large at the transition. Under an applied voltage, the resistance drops due to Joule heating. The relation between the applied power, V^2^/R, and the average resistance of the sample, R, may be expressed as: d(V^2^/R)/dT = 2(V/R)(dV/dT)−(V/R)^2^dR/dT > 0. Since dR/dT < 0, V may reach a maximum (dV/dT = 0) for high enough currents: the so-called current-controlled CC-NDR regime is reached. In the case of positive temperature coefficient (PTC) of resistance, a voltage-controlled VC-NDR regime may be reached beyond a maximal current. It can be shown that when the Newton law of cooling may be applied (V^2^/R∝ ΔT = T − T_0_, where T_0_ is the ambient temperature) and the activated nature of the resistance may be approximated by R = R(T_0_) exp(ΔT/T_1_), then ΔT(V_max_) = T_1_. For the ambient temperature of 295 K and Δ = 0.46 eV, T_1_ = ΔT ≈ 16 K, which is far below the transition. The result of this rough approximation indicates that the onset of the mixed state of switching VO_2_ crystals occurs within the CC-NDR regime. It can be easily shown that for R_L_ >> R (constant current conditions), all I(V) points in the CC-NDR regime may be reached under steady state conditions. For low R_L_, the NDR regime becomes unstable and the I–V characteristic jumps between two steady states connected by a line with slope dI/dV = (R_L_)^−1^ (e.g., Figure 2).
